# Microwave Ablation of Very-Early- and Early-Stage HCC: Efficacy Evaluation by Correlation with Histology after Liver Transplantation

**DOI:** 10.3390/cancers13143420

**Published:** 2021-07-08

**Authors:** Laura Crocetti, Paola Scalise, Elena Bozzi, Daniela Campani, Piercarlo Rossi, Rosa Cervelli, Irene Bargellini, Davide Ghinolfi, Paolo De Simone, Roberto Cioni

**Affiliations:** 1 Division of Interventional Radiology, Azienda Ospedaliero Universitaria Pisana, 56126 Pisa, Italy; paola.scalise@ao-pisa.toscana.it (P.S.); elenabozzi@libero.it (E.B.); piercarlo.rossi@ao-pisa.toscana.it (P.R.); rosa.cervelli@ao-pisa.toscana.it (R.C.); i.bargellini@ao-pisa.toscana.it (I.B.); r.cioni@ao-pisa.toscana.it (R.C.); 2 Department of Surgical, Medical and Molecular Pathology and Critical Care Medicine, University of Pisa, 56126 Pisa, Italy; daniela.campani@med.unipi.it (D.C.); paolo.desimone@unipi.it (P.D.S.); 3 Division of Pathology, Azienda Ospedaliero Universitaria Pisana, 56126 Pisa, Italy; 4 Division of Hepatobiliary Surgery and Liver Transplantation, Azienda Ospedaliero Universitaria Pisana, 56126 Pisa, Italy; d.ghinolfi@ao-pisa.toscana.it

**Keywords:** hepatocellular carcinoma, ablation, microwave (MW), liver transplantation, rad-path correlation

## Abstract

**Simple Summary:**

Microwave (MW) ablation is increasingly used worldwide as a therapeutic option in very-early- and early-stage hepatocellular carcinoma (HCC). Up to now, there have been few published studied showing the correlation with pathology in ablated nodules. The aim of our study was to retrospectively correlate the necrosis obtained with MW ablation with histological findings in excised livers from the time of liver transplantation to assess the MW ablation effectiveness. We obtained, in a population of 30 patients with 36 nodules, a complete ablation at 1-month imaging in 30/36 nodules (83.3%). At pathology, of the 36 treated nodules, 28 (77.8%) showed complete necrosis, and 8 (22.2%) showed partial necrosis. Good agreement was found between the imaging performed 1-month after treatment and the complete pathological response. These data confirm the effectiveness of MW ablation to percutaneously treat HCC nodules smaller than 3 cm with a high sensitiveness of radiological imaging in detecting a complete response after ablation.

**Abstract:**

Microwave (MW) ablation is a worldwide-diffused technique for the percutaneous ablation of hepatocellular carcinoma (HCC). Nevertheless, the efficacy of this technique still needs to be confirmed in pathological specimens. The purpose of this study was to evaluate the efficacy of MW ablation by correlation with histology in excised liver samples at the time of liver transplantation (LT). All patients with MW-ablated HCC who subsequently underwent LT between 2012 and 2020 were retrospectively evaluated. In the explanted livers, the treated lesions were evaluated at pathology, and the necrosis was classified as complete or partial. Thirty-six HCCs were ablated in 30 patients (20.9 ± 6.1 mm, a range of 10–30 mm). Ablations were performed with a single insertion of a MW antenna under ultrasound or CT guidance. A complete radiological response was demonstrated in 30/36 nodules (83.3%) in 24/30 patients (80%) at imaging performed one-month after MW ablation. At pathology, of the 36 treated nodules, 28 (77.8%) showed a complete necrosis, and 8 (22.2%) showed a pathological partial necrosis. Good agreement was found between the imaging performed one-month after treatment and the complete pathological response (Cohen’s k = 0.65). The imaging accuracy in detecting a complete response to treatment was 88.9%. All lesions with complete necrosis did not show recurrence at follow-up imaging until transplantation. The rad-path correlation in the explanted livers showed that MW ablation achieved a high rate of complete necrosis if a macroscopical complete ablation was obtained.

## 1. Introduction

Percutaneous treatment of hepatocellular carcinoma (HCC) by means of radiofrequency (RF) has been introduced in clinical guidelines as a potentially curative treatment, since 2005 [1], and the technique is widely available at most treatment centers. However, in the last decade, microwave (MW) ablation has been increasingly applied in clinical practice. Pre-clinical studies highlighted, as potential advantages of MW-based ablation, the ability to achieve higher temperatures (>100 °C) and larger ablation zones in shorter times, with less susceptibility to blood flow-induced heat sink effects [2,3]. 

The current evidence does not support the superiority of MW ablation compared to RF ablation in terms of patient overall survival (OS) [4]. Alternatively to survival analysis, the correlation between radiological findings after ablation and histopathological findings on the excised liver at the time of liver transplantation (LT) has been used to evaluate MW ablation effectiveness. However, radiological-pathological correlation studies conducted in HCC patients undergoing RF ablation and, subsequently, LT have shown inconsistencies between the radiologically assessed post-treatment response and pathological findings [5,6,7,8,9]. 

In particular, radiological evaluation overestimated the complete therapeutic success of RF ablation, which was in the range of 70–99%, while a complete pathological response was demonstrated in 55–72% of HCC nodules [8,9,10]. Limited evidence is available regarding MW ablation efficacy evaluation by means of radiological-pathological correlation. Therefore, the purpose of this retrospective study is to evaluate the effectiveness of MW ablation in obtaining a complete necrosis of HCC nodules by correlation with histological findings in patients who subsequently underwent LT.

## 2. Materials and Methods

### 2.1. Patients Population

Our prospective LT database was queried to identify HCC patients transplanted between 2012 and 2020 and, among them, the ones who were submitted to MW ablation before LT. All patients underwent at least one computer tomography (CT) or magnetic resonance (MR) evaluation before transplantation.

At our Center, enlisting for LT was performed according to the Up-to-Seven criteria for HCC in cirrhosis and AFP levels lower than 400 ng/mL [11]. After multidisciplinary tumor board evaluation, percutaneous MW ablation was offered to patients according to the following criteria: (a) up to three nodules < 3 cm; (b) lesion location farther than 1 cm from the gallbladder and/or the main biliary tree; (c) needle path to the lesion through non tumoral tissue; (d) platelet count > 50,000/µL and INR < 1.5; and (e) the absence of clinically relevant ascites. 

All included patients were then subdivided into two groups: in group (A), ablation was performed as bridge-to-transplant, while, in group (B), LT was offered due to HCC recurrence in the nodule treated by previous MW ablation or elsewhere in the liver. Before ablation, all patients underwent abdominal contrast-enhanced CT and/or dynamic contrast-enhanced MR imaging, and HCC was diagnosed according to typical hallmarks at imaging [12]. Confirmation of the diagnosis with a biopsy was required in case of nodules larger than 1 cm in size without typical radiological hallmarks [12]. Patients treated with trans-arterial chemoembolization or a combined approach (MW ablation plus transcatheter arterial chemoembolization) were excluded from the analysis.

### 2.2. MWA Technique

In all cases, ablations were performed with the same MW ablation system, HS AMICA (HS Hospital Service, Rome, Italy). The main features of the MW ablation system used for this study included:

- a 2450 MHz operating frequency;
- 140 W maximum continuous waves power at the generator; and
- straight, internally cooled 14 G or 16 G antennas, with built-in reflected waves suppressor (“mini-choke”).

The following technical parameters provided by the generator were recorded for all the procedures:

- power (P) released by the generator, expressed in watts (W); and
- ablation time (T), defined as the duration of ablation and expressed in seconds (s).

All the procedures were performed by two operators; the senior physician (with 9 years’ experience in ablation at the beginning of the study) assisted the junior in the initial 3 years of practice to ensure all the procedures were performed according to the standard of care. 

### 2.3. Post Procedural Imaging

One month after the procedure, all patients underwent contrast-enhanced CT and/or dynamic contrast-enhanced MR and followed-up with the same protocol every three months thereafter. Post-procedural imaging evaluation performed four weeks after ablation was used to compare the radiological and histological findings.

All imaging studies were conducted at our Institution. At CT, liver assessment was accomplished by using a quadriphasic protocol encompassing a 2.5-mm interval and axial images obtained in unenhanced, late arterial, portal venous and equilibrium phases. Iodinated contrast media (320–400 mgI/mL) was injected at 3–4 mL/min after the bolus test. At MR, liver assessment was accomplished by T1- and T2-weighted sequences, diffusion-weighted imaging and T1-weighted contrast enhanced sequences after the intravenous administration of Gd-EOB-DTPA or Gd-BOPTA. Imaging was retrospectively reviewed by two expert radiologists, and mRECIST criteria on the target lesion were applied [13]. 

In case of disagreement, the final mRECIST assessment was obtained from a direct consultation between the two specialists. A complete response (CR) was defined as the disappearance of any enhancement of the target lesion, while a partial response (PR) was defined as at least a 30% decrease in the sum of the diameters of viable (enhancement in the arterial phase) target lesions. Progressive disease (PD) was defined as at least a 20% increase in the sum of the diameters of viable target lesions and features classifiable as neither PR nor PD were defined as stable disease (SD) [13]. 

### 2.4. Pathological Assessment

After LT, gross and microscopic examination of the explanted livers was performed by one experienced pathologist. As an institutional policy, the gross examination of livers was performed after cutting each specimen into 5-mm slices and fixed in 10% formalin. All macroscopically identified suspected neoplastic nodules as well as all treated nodules were sectioned and subjected to standard hematoxylin-eosin staining. All the nodules were measured, and their location was assessed on the basis of the Coinaud’s classification system [14].

All the identified nodules were microscopically examined and classified, according to the International Consensus Group for Hepatocellular Neoplasia [15]. In all HCCs, microvascular invasion was evaluated, and tumor differentiation was graded on the basis of the Edmonson and Steiner classification [16]. For the treated nodules, complete necrosis was defined as the absence of viable tissue in the site of ablation. On the other hand, partial necrosis was defined if HCC cells were found within the nodule or nearby.

### 2.5. Statistical Methods and Data Availability

Categorical variables are reported as the number of cases and percentages; continuous variables are reported as the mean and standard deviation. The concordance between radiological imaging performed four weeks after treatment and pathological examination was assessed using Cohen’s k (https://idostatistics.com/cohen-kappa-free-calculator/ accessed on 2 June 2021). The agreement was considered excellent if k > 0.80; good if 0.60 < k < 0.80; moderate if 0.40 < k < 0.60; and insufficient if k < 0.40.

The diagnostic accuracy of imaging in defining CR was assessed by measuring the sensitivity, specificity, positive predictive values (PPV) and negative predictive values (NPV) (https://www.medcalc.org/calc/diagnostic_test.php accessed on 2 June 2021).

## 3. Results

### 3.1. Patients

A total of 32 patients and 38 HCC nodules were included in the study. Among them, two patients were then excluded from further analysis because of nodule dimension >30 mm and multiple bridging treatments on the treated nodule. Therefore, our final cohort consisted of 30 patients and 36 HCC nodules. The majority of them were male (93.3%), and the median age was 58.8 years ± 7.6 (range: 39–70 years). Hepatitis C was the main etiology for liver disease (66.6%). Five patients were also co-infected with HBV, while associations with alcohol consumption and autoimmune disorder were present in seven and one cases, respectively. The characteristics of the patients are summarized in Table 1. Confirmation of diagnosis with biopsy was not required in any case.

### 3.2. Ablation Protocol, Safety and Efficacy at Histology

An overall number of 36 nodules of HCC (mean dimension: 20.9 mm ± 6.1 mm, range 10–30 mm) were percutaneously ablated. Group A included 22 patients, while group B comprised 8 patients with new HCC nodules (*n* = 4), disease persistence/recurrence (*n* = 2) and new HCC nodules plus disease persistence/recurrence (*n* = 2). Twenty-six patients (86.7%) had only one HCC nodule while 4 patients had multinodular HCC (13.3%), with a mean number of 1.2 nodules ablated per patient.

All ablations were performed with a single insertion of the MW antenna (14 G in 19 nodules, 16 G in 17 nodules). Ablation was performed under ultrasound guidance in most cases (33/36, 91.7%), while CT guidance was preferred in three cases (3/36, 8.3%). All patients were treated in a single session. The MW power and ablation time ranged from 30 to 60 W and 120 to 600 s, respectively. The time interval between ablation and transplantation ranged from 1 to 73 months (mean 16.4 months).

Neither major complications nor neoplastic seeding along the needle path occurred. Minor complications after ablation included mild perihepatic fluid that did not require treatment in three patients (10%); and bland segmental portal vein thrombosis shown at the one-month CT scan (3.3%), although without any evidence reported at pathology after LT. The median length of hospital stay was 2 days (range 1–3 days).

**Figure 1:**
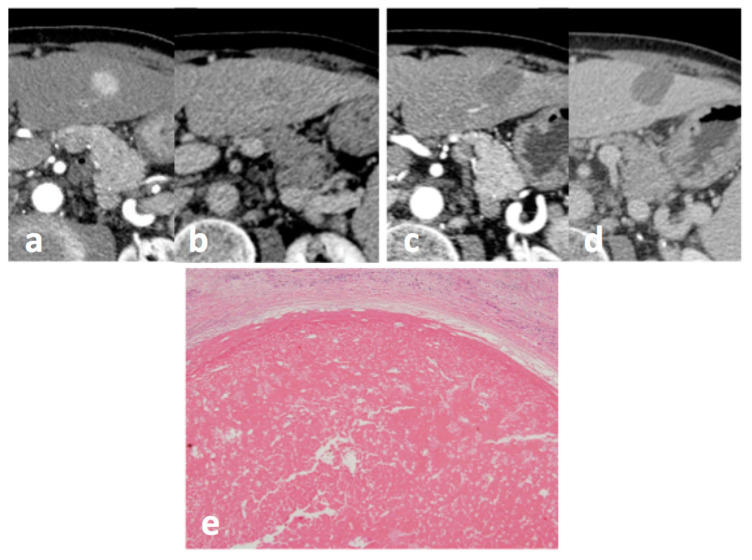
Small HCC in hepatic segment 3 at CT: before treatment, (**a**) arterial phase and (**b**) portal-venous phase, and 1 month after treatment with MW ablation (**c**) arterial phase, and (**d**) portal-venous phase: no enhancing tissue is depicted, and the response was classified as CR according to mRECIST. A complete pathological response was confirmed at pathology: (**e**) HE staining (magnification 10×).

At post-procedural imaging performed one-month after MW ablation, a complete radiological response was demonstrated in 30/36 nodules (83.3%), while viable disease on the treated nodule was evidenced in the remaining 6/36 nodules (16.7%). At pathology, of the 36 treated nodules, 28 (77.8%) showed a complete necrosis, and 8 (22.2%) showed a pathological partial necrosis (Figure 1). The presence of microscopic vascular invasion was demonstrated in 5/36 nodules (13.9%). No cases of macrovascular invasion were detected as well as metastatic nodal involvement at the hepatic hilum.

The relationship between imaging performed one month after treatment and a complete pathological response is shown in Table 2. Cohen’s k was 0.65, indicating good agreement. The diagnostic accuracy of CT and MR in detecting a complete response to treatment was 88.9%. Of the 30 nodules with a complete radiological response, 27/30 (90%) showed complete necrosis, and 3/30 (10%) showed partial necrosis on pathological analysis. Of the six nodules with a persistent viable tumor at imaging, viable tissue was not confirmed in one nodule at pathology. After a retrospective revision of the imaging, the presence of transient hepatic attenuation difference near the nodule was assessed.

Among the three lesions with a one-month complete radiological response and partial necrosis at histological examination, two showed pathological micro-angioinvasion. All lesions with pathological complete necrosis did not show any suspect findings of recurrence at follow-up imaging until transplantation.

When partial necrosis was found in cases with negative imaging findings at one- month, macroscopic disease recurrence was noticeable at imaging 21 months after ablation in one case, appearing as viable solid tissue peripherally to the treated lesion with enhancement in the CT arterial phase and washout in the venous and late phases. In the other two ablated lesions with vital tissue on histology, no recurrence was highlighted at imaging until the last evaluation before transplantation performed 1 and 3 months before transplantation (Table 2). 

## 4. Discussion

Among locoregional treatments, RF ablation is considered as potentially curative in the setting of cirrhotic patients with very-early- and early-stage HCC by the EASL guidelines [12]. In patients with very-early-stage HCC, RF ablation in favorable locations can be adopted as a first-line therapy even in surgical patients. For a single tumor of 2 to 3 cm in size, RF ablation is an alternative to surgical resection based on technical factors (location of the tumor) and hepatic and extra-hepatic patient conditions [12]. In the same guidelines, MW ablation is considered as a promising technique for its results in terms of local control and survival. The level of evidence is low and the available data were not sufficient to foresee an established role of MW ablation in the guidelines.

In some centers, RF ablation has been replaced over the years by MW ablation. This change in clinical practice is due to the pre-clinical evidence that, with a microwave apparatus, it is possible to obtain higher temperatures in comparison with RF (>100 °C), in considerably shorter times (few minutes). In addition, it is well known that MW are less influenced by the “heat sink effect”, which is the main reason for tumor local recurrence [2,3,17,18,19].

In addition, antennas for MW are easy to use, and they allow obtaining large tumor ablation with only one insertion and a short ablation time [20]. It could be possible, that, due to these features, an increased number of patients are actually treated with MW ablation with respect to RF, where electrodes with complex designs or multiple electrodes are needed to obtain large volumes of necrosis. Currently, cohort studies and some randomized controlled studies comparing MW ablation and RF ablation have been published. The results of cohort studies demonstrated that there are some advantages in the use of MW technology in terms of local recurrence, in particular in the treatment of nodules larger than 3 cm [21,22].

On the other hand, the small number of published randomized studies failed to show a statistically significant superiority of MW ablation in comparison to RF ablation in terms of the overall survival and disease-free survival [23,24,25]. The superiority of MW ablation with respect to RF ablation was not demonstrated in several metanalyses [4,26,27,28].

In 2020, a study comparing RF ablation and MW ablation by using a “propensity score matching” analysis to evaluate the prognostic factors associated with disease progression after percutaneous ablation was published. The authors concluded that the percentage of local tumor progression (LTP) was lower after MW in comparison to RF independently from tumor dimension and the vicinity to vascular structures, and RF ablation was identified as an independent predictor for LTP, even in lesions up to 30 mm. [29].

Due to these premises, the conduction of a randomized controlled trial aimed at demonstrating the superiority of MW ablation versus RF ablation in term of OS would likely require a huge number of patients with, consequently, very high costs. A valid approach to assess the real efficacy of MW ablation is, therefore, to directly evaluate the effect of MW ablation on the surgical specimen or on the excised liver at the time of LT. This approach allows for an objective and reliable assessment of the therapeutic effect.

In our series, 78% of all HCC nodules treated with MW ablation had complete necrosis, in one session. This figure is higher than those previously reported in series using RF ablation and rad-pathology correlation in explanted livers [8,9,10].

In particular, in both studies of Lee et al. [9] and Serra et al. [8], who treated all patients in one, two or three RF ablation sessions, the percentages of complete necrosis were reported as 72% and 62%, respectively. In our series, patients with persistence after one MW session were subsequently submitted to transplantation, and additional MW ablation procedures were not performed. Therefore, MW ablation seems to have obtained results at least comparable to those obtained with RF ablation but in one treatment session and with clinical and economic advantages. In patients outside transplantation settings, a repeat MW session could allow reaching a complete response.

In our study, the imaging showed a high sensitivity to depict the presence of a complete response soon after ablation at 1 month. Of the 30 nodules with a complete radiological response, 27/30 (90%) showed complete necrosis and, only in one case, were the imaging features suspected for disease persistence not confirmed at pathology.

This is likely due to the peculiar ablation mechanism of MW, which is able to reach rapidly higher temperatures, due to active tissue heating. On the contrary, with RF ablation, cytotoxic temperatures are reached slowly and mainly through passive heating. This may cause the development of more evident inflammation areas, particularly at the ablation margins that correspond at imaging to the hypervascular rim in the arterial phase, and this appearance could be misinterpreted as the presence of a viable tumor [30].

In 3 out of 36 nodules (8.3%) classified at imaging as a complete response, foci of a viable tumor were present at the pathological examination. In one case, macroscopic disease recurrence was noticeable at imaging 21 months after ablation. The volume of residual vital tissue was likely too low to be depicted by imaging. In the other two lesions with vital tissue on histology, no recurrence was highlighted at imaging until the last evaluation before transplantation, and only peripheral small foci of vital cells were demonstrated at histology.

Viable tissue was not confirmed at pathology in one of the six nodules with persistent viable tumor at imaging. At the retrospective imaging revision, the presence of transient hepatic attenuation difference near the nodule was assessed and considered responsible for the imaging misinterpretation. A good correlation (Cohen’s k = 0.65) between imaging and pathological examination was obtained, different from the data reported by Serra et al. [8] where it was moderate (Cohen’s k = 0.48) [8].

The safety of MW ablation as a neo-adjuvant therapy before liver transplantation was also confirmed. All the procedures were performed according to the standard of practice [19], and neither major complications nor neoplastic seeding along the needle path occurred. Progression with macrovascular invasion was never detected and neither was metastatic nodal involvement at the hepatic hilum. 

Our study has certain limitations. First, the number of treated patients and included nodules was exiguous. Nevertheless, this is the first series with a rad-path correlation of MW ablation results in subsequently transplanted patients. Second, the interval time between ablation and liver transplant was very variable. In the majority of cases, ablation was performed with “bridge” intent, while, in a smaller number of cases, the main indications for transplant was represented by the persistence/local recurrence or the presence of new HCC lesions. 

Third, the procedures were performed under ultrasound or CT guidance. The ablation protocol was planned according to the manufacturer’s ablation chart and physician’s experience in order to provide an ablation volume that included a margin. An immediate check of the adequacy of ablation margins, by means of dedicated systems, was not performed. This could improve the results of MW ablation by increasing the rates of complete radiological and pathological responses.

## 5. Conclusions

In our experience, MW ablation performed well in obtaining valid tumor local control and is a useful tool in bridging to transplantation, since no rapid progression of HCC, tumor insemination or macrovascular invasion was observed. MW ablation treatment allowed obtaining results that were slightly superior to RF ablation in a single treatment session (a complete pathological response in 78% of HCC nodules) and with reliable imaging findings (diagnostic accuracy: 89%). In the future, cost-effectiveness assessments could bring new evidence to support the increasingly widespread use of MW ablation.

## Figures and Tables

**Table 1:** The demographic, procedural and pathological data.

Variable			*n* (%) or Mean (±SD)
**Demographics**			
Number of patients		Total	30
Sex		M/F	28 (93.3%)/2 (6.7%)
Age (years, range)			58.8 ± 7.6 (39–70)
Etiology	Infectious	HCV	20/30 (66.6%)
		HBV	5/30 (16.7%)
	Alcoholic		7/30 (23.3%)
	Autoimmune		1/30 (3.3%)
Child-Pugh	At diagnosis/At LT	A	30 (100%)/30 (100%)
Mean time ablation-LT (months, range)			16.4 (1–73)
Number of HCC nodules			36
HCC nodule mean diameter (mm, range)			20.9 mm ± 6.1 mm (10–30 mm)
Single HCC nodule			26/30 (86.7%)
Multiple HCC nodules			4/30 (13.3%)
Mean number of ablated HCC nodules per patient			1.2
**MW Ablation Procedure**			
Imaging guidance	Ultrasound		33/36 (91.7%)
	CT		3/36 (8.3%)
Gauge	14		19/36 (52.8%)
	16		17/36 (47.2%)
Time (seconds, range)			120–600 s
Power (watt, range)			30–60 watt
Complications	Perihepatic ascites		3/30 (10%)
	Portal vein thrombosis		1/30 (3.3%)
**One-Month Post Procedural Imaging Evaluation**			
Complete response			30/36 (83.3%)
Viable disease			6/36 (16.7%)
**Pathological Data**			
Complete necrosis			28/36 (77.8%)
Partial necrosis			8/36 (22.2%)
Vascular invasion	Macroscopic/microscopic		0/36 (0%)/5/36 (13.9%)
Lymph-node metastasis at hepatic hilum			None

**Table 2:** Rad-path correlation.

Imaging (CT/MR) *	Pathology	
	CPR	No CPR
**CR**	*n* = 27	*n* = 3
**PR**	*n* = 1	*n* = 5
**Cohen’s k**	0.65	
**Statistics**	**Value**	**95% CI**
Sensitivity	96.43%	81.65–99.91%
Specificity	62.50%	24.49–91.48%
Positive predictive ratio	90.00%	78.58–95.67%
Negative predictive ratio	83.33%	40.40–97.36%
Diagnostic accuracy	88.89%	73.94–96.89%

* Performed 1 month after treatment; CPR: Complete pathological response; CR: Complete response; and PR: Partial response.

## Data Availability

Data are available on request from the corresponding author due to privacy restrictions.
